# Special Announcement

**Published:** 1907-05

**Authors:** 


### Journal-Record of Medicine

Successor to Atlanta Medical and Surgical Journal, Established 1855, and Southern Medical Record, Established 1870.

OWNED BY THE ATLANTA MEDICAL JOURNAL CO.

Published Monthly.

*Official Organ Fulton County Medical Society, State Examining Board, Presbyterian Hospital, Etc.*

VOL. IX. MAY, 1907. No. 2

BERNARD WOLFF, M. D., EDITOR, 319-320 Prudential Bldg.

GEORGE BROWN, M. D., BUSINESS MANAGER, Business Office 1½-5½ S. Broad St.

E. G. BALLENGER, M. D., ASSOCIATE EDITOR AND ASSISTANT BUSINESS MANAGER.

### ORIGINAL COMMUNICATIONS

### SPECIAL ANNOUNCEMENT.

Since the last issue, the Journal-Record has undergone a change in business administration and intends in the future to pursue a somewhat different policy as to the character of its contents.

Dr. M. B. Hutchins, who has been associated with the Journal-Record as business manager since its establishment as the result of the union of the Atlanta Medical and Surgical Journal and the Southern Medical Record, has parted with his interest, and the business affairs of the publication have passed into other hands. The Journal-Record is pleased to announce that it now owns its own plant, thus insuring for itself the permanence and stability which comes from such a possession. It will in the future be issued from its own press and the endeavor will be made to present each month a highly creditable product of the printer's art.

We have long entertained the view that the average medical journal was conducted in a manner not well suited to the ordinary subscriber. The papers appearing as original matter are too often highly technical, of too restricted an interest, or too abstruse and abstract to be adapted properly to the needs of the hard-working, practical physician. Such publications are not in close touch with those who support it and one remains as a subscriber from courtesy, or association or from some other motive other than a sense of helpful indispensability. It is the desire of the Journal-Record to alter its policy in this respect and to seek to be of real assistance to those who are kind enough by their subscriptions and interest to keep it afloat. To this end, it wishes to urge its friends to display their interest and test its useulness by contributing to its columns at frequent intervals, or occasionally, reports or experiences trom their practice. If such reports occupy only a few paragraphs, they are entirely acceptable and may furnish opportune aid to some reader.

Frequently a subscriber remote from professional counsel is confronted with some perplexing clinical problem—some case where a word of advice or an opinion would be illuminating. We wish to help in such a condition.

We have arranged with some of the foremost practitioners in the State, covering every branch of medicine, to give their opinion upon cases submitted to them by a careful description. Write an account of your case as clearly as possible, and we shall take pleasure in referring it to the proper person for an opinion. These reports will be published in the Journal-Record with all promptness possible. This will constitute a sort of post-graduate course and we invite all of our readers to participate.

Many physicians are deterred from reporting their cases from inexperience in writing, but this need deter no one. Whoever can think, can write—the method or the manner is of small consequence. The chief consideration is to bring out the question to be answered or the fact to be recorded. The editor will reserve the usual editorial privilege to "lick into shape" such copy as appears to require it.

This announcement is no empty bluff. We wish to aid our friends, and we want it reciprocal.

If you have any case in the whole range of ills, which you wish to report or advise over, from baldness to callused heel, write us about it and the best brains in the profession in Georgia are ready to help you out of your difficulty.

